# Effect of Physical Exercise on Telomere Length: Umbrella Review and Meta-Analysis

**DOI:** 10.2196/64539

**Published:** 2025-01-10

**Authors:** Juan Luis Sánchez-González, Juan Luis Sánchez-Rodríguez, Rogelio González-Sarmiento, Víctor Navarro-López, Raúl Juárez-Vela, Jesús Pérez, Javier Martín-Vallejo

**Affiliations:** 1 Faculty of Nursing and Physiotherapy, Department of Physiotherapy, University of Salamanca, Salamanca, Spain; 2 Faculty of Psychology, Department of Basic Psychology, Psychobiology and Methodology, University of Salamanca, Salamanca, Spain; 3 Faculty of Medicine, Department of Medicine, University of Salamanca, Salamanca, Spain; 4 Faculty of Health Sciences, Department of Physical Therapy, Occupational Therapy, Rehabilitation and Physical Medicine, Universidad Rey Juan Carlos, Madrid, Spain; 5 Faculty of Health Sciences, Department of Nursing, University of La Rioja, Logroño, Spain; 6 Faculty of Medicine, Department of Psychiatry, University of Salamanca, Avenida Donantes de Sangre s/n, Salamanca, 37007, Spain, 34 7535596578; 7 Department of Psychiatry, University of Cambridge, Cambridge, United Kingdom; 8 Cambridgeshire and Peterborough NHS Foundation Trust, Cambridge, United Kingdom; 9 Norwich Medical School, University of East Anglia, Norwich, United Kingdom; 10 Faculty of Medicine, Department of Statistics, University of Salamanca, Salamanca, Spain

**Keywords:** aging, chromosome, exercise, meta-analysis, telomere, telomerase, genes, genome, DNA

## Abstract

**Background:**

Telomere length (TL) is a marker of cellular health and aging. Physical exercise has been associated with longer telomeres and, therefore, healthier aging. However, results supporting such effects vary across studies. Our aim was to synthesize existing evidence on the effect of different modalities and durations of physical exercise on TL.

**Objective:**

The aim of this study was to explore the needs and expectations of individuals with physical disabilities and their interventionists for the use of a virtual reality physical activity platform in a community organization.

**Methods:**

We performed an umbrella review and meta-analysis. Data sources included PubMed, Embase, Web of Science, Cochrane Library, and Scopus. We selected systematic reviews and meta-analyses of randomized and nonrandomized controlled clinical trials evaluating the effect of physical exercise on TL.

**Results:**

Our literature search retrieved 12 eligible systematic reviews, 5 of which included meta-analyses. We identified 22 distinct primary studies to estimate the overall effect size of physical exercise on TL. The overall effect size was 0.28 (95% CI 0.118-0.439), with a heterogeneity test value Q of 43.08 (*P*=.003) and *I*² coefficient of 51%. The number of weeks of intervention explained part of this heterogeneity (Q_B=8.25; *P*=.004), with higher effect sizes found in studies with an intervention of less than 30 weeks. Exercise modality explained additional heterogeneity within this subgroup (Q_B=10.28, *P*=.02). The effect sizes were small for aerobic exercise and endurance training, and moderate for high-intensity interval training.

**Conclusions:**

Our umbrella review and meta-analysis detected a small-moderate positive effect of physical exercise on TL, which seems to be influenced by the duration and type of physical exercise. High quality studies looking into the impact of standardized, evidence-based physical exercise programs on TL are still warranted.

## Introduction

Life expectancy has increased worldwide over the last century. The United Nations World Population Prospects [1] estimates that the world’s population aged 65 years or older will rise by 16% in 2050, doubling the number of children younger than 5 years. This prospect will lead to a significant increase in age-related illnesses, such as cancer, dementia, or cardiovascular diseases [2], and is prompting research into the primary mechanisms of aging and, subsequently, strategies to promote a healthy late-life.

López-Otín et al [3] have recently postulated 12 basic biological mechanisms of aging: epigenetic alterations, loss of proteostasis, disabled macroautophagy, deregulated nutrient-sensing, mitochondrial dysfunction, cellular senescence, stem cell exhaustion, altered intercellular communication, chronic inflammation, dysbiosis, genomic instability, and telomere attrition. Indeed, one of the main theories that has attempted to explain aging relates to telomere length (TL) and the role of telomerase, an enzyme responsible for maintaining and elongating telomeres. Telomeres are essential nucleoprotein structures located at the termini of eukaryotic chromosomes that play pivotal roles in safeguarding genomic integrity and regulating processes such as tumor suppression and aging [4]. Typically composed of a repetitive guanine-rich sequence extending from the chromosome end in the 5’ to 3’ orientation, paired with a complementary cytidine-rich strand [5], telomeres exhibit variations in sequence among species, yet share a common repetitive pattern across organisms. Measuring around 15 kilobases at birth in human somatic cells, telomeres undergo gradual attrition, with approximately 25 to 200 bases lost from their ends during each cell division in the absence of telomerase [6]. Upon reaching a critical length, telomeres signal cell cycle arrest, leading to cellular senescence and eventual demise [7]. Telomeres serve to shield chromosome ends from degradation and fusion, thereby upholding genomic stability [8,9].

There are different demographic factors that influence TL, such as genetics that may explain the high heritability of TL [10,11]; sex, with longer telomeres found in adult females [12]; or ethnicity, with the White community usually having longer telomeres than Black or Hispanic communities [13]. Also, stress levels have been associated with shorter TL, which may be due to oxidative stress and reduced telomerase enzyme activity [14,15]. Obesity [16], alcohol consumption [17], and smoking [16,18] are also factors that negatively influence TL.

Notably, longer telomeres have been found in people who exercise regularly [19] or with higher levels of daily physical activity [20,21]. Indeed, one of the most studied interventions regarding TL is physical exercise. Recent systematic reviews and meta-analyses [22-26] have investigated the effect of physical exercise on the TL of clinical and nonclinical samples, showing some positive, but still inconclusive, results. Aerobic exercise, such as running or swimming, has been consistently associated with longer telomeres. Denham et al [27] conducted a study that found individuals who regularly engage in aerobic exercise show greater telomerase activity, the enzyme that helps maintain TL, compared with those who lead a sedentary lifestyle. In addition, Ludlow et al [28] demonstrated that resistance training, which includes exercises like weight lifting, may also have beneficial effects on TL, possibly through mechanisms involving the reduction of oxidative stress and inflammation. In addition, more recent research has indicated that high-intensity training, such as high-intensity interval training (HIIT), may be more effective in preserving TL compared with low- to moderate-intensity exercises [29]. This type of exercise can induce more robust adaptive responses at the cellular level, including increased expression of genes related to longevity and telomere protection [3].

The aim of this umbrella review was to synthesize existing evidence on the effect of different modalities and durations of physical exercise on TL to inform the implementation of evidence-based physical exercise programs or recommendations to add healthier years into longer lives.

## Methods

### Overview

This umbrella review was conducted in accordance with the PRISMA (Preferred Reporting Items for Systematic Reviews and Meta-Analyses) guidelines [30], and it is registered in the International Prospective Register of Systematic Reviews (PROSPERO; registration number CRD42024500736).

### Inclusion Criteria

The inclusion criteria and description of studies in this review followed the PICOS (Population, Intervention, Control, Outcomes, and Study design) framework for reviews [31].

#### Study Design

We selected systematic reviews, with or without meta-analyses, of randomized and nonrandomized controlled clinical trials, excluding nonexperimental designs, such as observational studies. No restrictions were applied on the basis of any particular language, following current international recommendations [32].

#### Population

Study participants were people with or without a clinical condition. The systematic reviews had to explicitly state that they included humans in their analyses. Therefore, studies involving experimental animals, such as rodents, were excluded.

#### Intervention and Control

We selected all systematic reviews and meta-analyses comparing the effects of physical exercise versus other intervention, or none, on TL. In addition, we further divided the results according to different types of physical exercise. If any reviews included primary studies combining different types of exercise, they were classified as “combined.”

#### Outcome Measure

The outcome measure was TL-related calculations.

### Search Strategy

We performed a literature search for scientific articles published until January 19, 2024, using the following databases: PubMed (Medline), PEDro, Embase, Web of Science, and Cochrane Library. Multimedia Appendix 1 provides the search strategies, tailored for each database. A total of 2 independent reviewers (JLS-G and JLS-R) conducted the search using the same methodology. Any discrepancies during this process were resolved through consensus including a third reviewer (VN-L). In addition, we manually examined the reference sections of the original studies, and, if needed, contacted the authors for additional information.

### Selection Criteria and Data Extraction

A total of 2 independent reviewers (JLS-G and JLS-R) initiated a screening process to evaluate relevance of the systematic reviews and meta-analyses. The initial screening was based on the title, abstract, and keywords for each review. When there was no consensus, or if the abstracts provided insufficient information, the full text was examined. During the second screening phase, the full text was evaluated to confirm inclusion criteria. Data presented in the results section were extracted using a protocol to ensure retrieval of the most relevant information for each study. The sample sizes, type of exercise, duration, means, and SDs of the telomere size data were extracted for each of the studies included in this review. During the analysis, we checked whether the results were repeated in different reviews or meta-analyses; if the effect sizes coincided, only one of the effect sizes was chosen; if the effect sizes did not match, the primary study was selected.

### Methodological Quality Assessment

A total of 2 independent reviewers (JLSG and VNL) evaluated the methodological quality of the systematic reviews and meta-analyses, using the modified quality assessment scale for systematic reviews (AMSTAR-2 [A Measurement Tool to Assess Systematic Reviews]). AMSTAR-2 [33] is a questionnaire comprised of 16 domains, with simple categorical options: “yes,” when the result is positive; “no,” when the standard is not met or there is not enough information to answer it; and “partial yes,” when there was partial adherence to the standard. In addition, we calculated the kappa coefficient (κ) and percentage (%) agreement scores to assess reliability before reaching consensus. Interrater reliability was estimated using κ, where κ>0.7 indicates a high level of agreement between reviewers, κ of 0.5‐0.7 indicates a moderate level of agreement, and κ<0.5 suggests a low level of agreement [34].

### Risk of Bias Assessment

We evaluated the risk of bias using the Risk Of Bias in Systematic reviews (ROBIS) assessment, which includes three domains: (1) relevance of assessment (optional); (2) identification of concerns with the review process through 4 domains related to study eligibility criteria, identification and selection of studies, data collection and study appraisal, and synthesis and findings; and (3) judgment on the risk of bias. The ROBIS tool includes signaling questions to assess specific domains and to guide the judgment of the systematic review’s risk of bias, with responses categorized as “yes,” “probably yes,” “probably no,” “no,” or “no information.” The risk of bias is then categorized as “low,” “high,” or “unclear” [35].

A total of 2 independent reviewers (JLS-G and JLS-R) assessed the risk of bias in the selected studies. In addition, we computed the kappa coefficient (κ) and percentage (%) agreement scores to evaluate reliability before reaching consensus.

### Grading of Evidence

The Physical Activity Guidelines Advisory Committee Grading Criteria (PAGAC) were used to evaluate grading of evidence. The criteria used for assessing the quality of evidence included (1) applicability of the study sample, exposures, and outcomes to the research question; (2) generalizability to the population of interest; (3) risk of bias/study limitations; (4) quantity and consistency of findings across studies; and (5) magnitude and precision of the effect. Based on this information, final evidence grades and conclusion statements for each research question were formulated [36].

### Overlap of Primary Studies

The overlap analysis of primary studies among the systematic reviews was performed with the Graphical Representation of Overlap for OVErviews (GROOVE) tool [37]. Using a matrix of evidence, GROOVE establishes the number of primary studies and systematic reviews, the absolute number of overlapped and nonoverlapped primary studies, and an overall corrected covered area (CCA) assessment. GROOVE also offers a detailed CCA assessment for each possible pair of systematic reviews (or “nodes”), with a graphical and easy-to-read representation of these results. In addition, it provides an optional function that incorporates the structural missingness within the matrix.

### Data Synthesis and Analysis

Given the high variability of designs, patients and endpoints across studies, results were integrated following a random effects model. The Hedges unbiased standardized mean difference was used to determine effect sizes. Heterogeneity was measured using Q-tests, *I*^2^ coefficients, and prediction intervals [38]. Metaregression and metapartition [39] were used to explain heterogeneity. These techniques involve partitioning sum of squares of the homogeneity test into 2 components, Q_B_ and Q_W_, to account for quantitative or qualitative variables. If the Q_E_ value was large and statistically significant compared with the value of Q_W_, it was considered that this variable explained part of the heterogeneity found in the integration of results across studies. If the variable was qualitative, subgroups of studies, which differed in the effect size and also showed less heterogeneity, were created. The procedure was repeated for subgroups that still presented large heterogeneity for other variables. Publication bias was assessed using the contour-enhanced funnel plot, Egger test, Doi plot, and LFK index procedures. 95% CIs were calculated for effect sizes. The significance level was set at 5%. The analyses were carried out with the *meta* v7.0, *metafor* v4.6 and *metasens* 1.5‐2 libraries of the R statistics software (version 4.4.0; R Foundation for Statistical Computing).

### Ethical Considerations

As this is an umbrella review, it is not necessary to have the approval of the ethics committee.

## Results

### Study Selection

**Figure 1.**
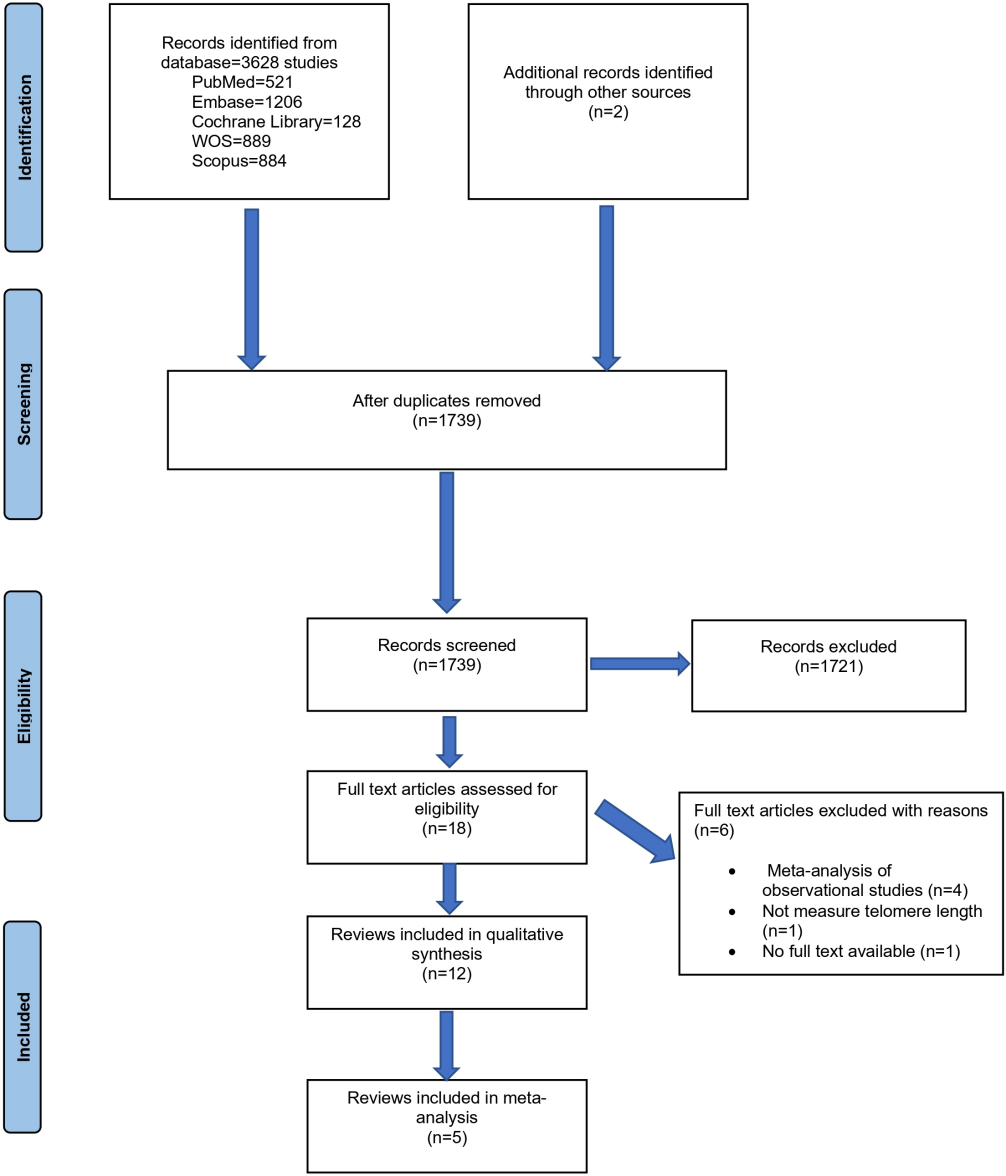
PRISMA (Preferred Reporting Items for Systematic Reviews and Meta-Analyses) flowchart.

The initial literature search revealed 3628 records. In addition, 2 more were retrieved manually from the references. A total of 12 systematic reviews were eligible for qualitative synthesis, and 5 of them allowed a meta-analysis. Figure 1 shows the study screening strategy.

### Characteristics of the Systematic Reviews

Table 1 lists the characteristics of the systematic reviews and meta-analyses (type of study, design, sample, intervention, lab technique to measure TL, risk of bias, evaluation of quality, and conclusions).

**Table 1.** Characteristics of the systematic reviews.

Study	Type of study	Study design, n		RCCTs^a^ or CCTs^b^ with physical exercise intervention					Risk of bias	Evaluation of quality	Conclusions
		Interventional	Observational	Sample		Intervention		Lab technique for TL^c^			
				n	Condition (number of studies, n)	Type (n)	Duration: weeks (n)				
Song et al [26]	Systematic review and meta-analysis	7 (RCCTs)	0	939	Healthy (3), breast cancer (2), polycystic ovary syndrome (1), and obese (1)	Aerobic exercise (4) and endurance training (3)	48 (2), 24 (3), and 16 (2)	qPCR^d^	Yes	No	The type and duration of exercise for positive improvement in TL is aerobic exercise for more than 6 months.
Buttet et al [22]	Systematic review and meta-analysis	13 (RCCTs) and 3 (CCTs)	5	908	Healthy (5), obese (1), and myocardial infarction (1)	Endurance training (7), strength (1), and HIIT^e^ (1)	48 (2), 32 (1), and 24 (4)	qPCR	No	No	A lifestyle intervention with physical activity + diet can increase TL, independently of population characteristics or baseline TL.
Sánchez-González et al [19]	Systematic review and meta-analysis	8 (RCCTs) and 1 (CT)	0	1320	Healthy (9)	Endurance training (4), aerobic exercise (4), HIIT (3), and aerobic + endurance (1)	8 (1), 16 (1), 24 (4), and 52 (3)	qPCR	Yes	Yes	The findings suggest that HIIT seems to have a positive effect on TL compared with other types of exercise such as endurance training or aerobic exercise in healthy population.
Valente et al [25]	Systematic review and meta-analysis	6 (RCCTs)	16 (case-control studies), 2 (prospective cohort), 3 (cross-sectional), and 3 (retrospective cohort)	612	Healthy (5) and obese (1)	Endurance training (5), strength (1), aerobic exercise (1), and HIIT (1)	56 (1), 48 (1), and 24 (4)	qPCR	Yes	No	There is very low certainty that physically active individuals have longer telomeres with a moderate effect, but this effect is probably overestimated.
Denham et al [23]	Systematic review and meta-analysis	12 (NR^f^)	0	487	Healthy (6) and chronic fatigue (1)	HIIT (1), aerobic exercise (3), Pilates training (1), and endurance training (2)	52 (1), 24 (2), 22 (1), 8 (2), and 1 (1)	qPCR	Yes	No	Exercise training as an inexpensive lifestyle factor that increases telomerase reverse transcriptase expression and telomerase activity. Regular exercise training could attenuate telomere attrition through a telomerase-dependent mechanism and ultimately extend health-span longevity.
Barragán et al [40]	Systematic review	12 (NR)	53 (cross-sectional studies), 13 (case-control), and 9 (longitudinal)	1.056	Healthy (12)	Endurance training (5), combined training (1), aerobic exercise (6), NR (1), and HIIT (1)	56 (1), 52 (2), 24 (3), 8 (2), 1 (2), and NR (2)	qPCR	No	Yes	Although fewer sedentary activities, optimal sleep habits, and non- or ex-smoker status have been associated with less telomere shortening, several methodological issues were detected, including the need for more targeted interventions and standardized protocols to better understand how physical activity and sleep can impact TL and aging.
Schellnegger et al [41]	Systematic review	8 (RCCTs) and 7 (CCTs)	27 (observational studies)	1.700	Healthy (13) and obese (2)	Aerobic exercise (8), endurance training (8), and HIIT (1)	1 (6), 6 (1), 8 (1), 12 (1), 24 (5), and 52 (2)	qPCR	No	No	Physical activity with regular aerobic training of moderate to vigorous intensity appears to help preserve TL.
Prathap et al [42]	Systematic review	2 (RCCTs): 1 in rodents	3 (literature review)	151	Breast cancer (1)	Aerobic exercise (1)	24 (1)	qPCR	No	No	Based on the evidence collected it can be suggested that chronic moderate intensity aerobic exercise in a lifelong practice shows beneficial effects in a dose-response manner in cancer prevention by modulating telomeres through epigenetic mechanism.
Quiao et al [43]	Systematic review	16 (RCCTs) and 14 (CCTs)	—^g^	562	Myocardial infarction (1), healthy adults (4), obese (1), and polycystic ovary syndrome (1)	Combined training (1), endurance training (3), and aerobic exercise (3)	8 (1), 12 (1), 16 (2), 20 (1), 24 (1), and 48 (1)	qPCR	No	Yes	Weight-loss and comprehensive lifestyle intervention strategies show encouraging impacts in delaying telomere shortening. More rigorous studies targeting populations at different age stages through life span are needed.
Min et al [44]	Systematic review	2 (RCCTs)	3 (cross-sectional study)	247	2 (breast cancer survivor)	Aerobic exercise (2)	24 (1) and 48 (1)	qPCR	No	No	Three of the five studies reported that physical activity has a significant relationship in delaying TL shortening, but others observed no association between physical activity and TL in breast cancer survivors.
Marques et al [45]	Systematic review	4 (RCCTs)	16 (cross-sectional)	647	Obese (1) and healthy (3)	Aerobic exercise (4), HIIT (1), and endurance training (1)	24 (3) and 48 (1)	qPCR	No	Yes	Better cardiorespiratory fitness or a large cardiorespiratory training load are associated with an increase in TL. Although, TL was related to regular moderate-to-vigorous aerobic exercise and cardiorespiratory fitness in older healthy humans, it was not related to cardiorespiratory fitness among young subjects.
Himbert et al [46]	Systematic review	10 (RCCTs) and 11 (CCTs)	—	439	Obese (1)	Aerobic exercise (1)	48 (1)	qPCR	No	No	The inconsistent effects of weight loss on telomere length or DNA repair suggest the need for a re-assessment of intervention designs and assay methodology to definitively address this topic.

a RCCT: randomized controlled clinical trial.

b CCT: (nonrandomized) controlled clinical trials.

c TL: telomere length.

d qPCR: polymerase chain reaction quantitative.

e HIIT: high-intensity interval training.

f NR: not reported.

g Not applicable.

### AMSTAR-2 Appraisal

The overall confidence ranged from critically low to moderate quality scores. A total of 2 studies (16.6%) obtained an overall confidence of moderate, and 10 studies (83.4%) had critically low quality (Multimedia Appendix 2). The items with the highest scores were “did the review authors use a comprehensive literature search strategy?”, “did the review authors describe the included studies in adequate detail?”, “did the review authors provide a satisfactory explanation for, and discussion of, any heterogeneity observed in the results of the review?”, and “did the review authors report any potential sources of conflict of interest, including any funding they received for conducting the review?” The lowest scoring items were “did the review authors account for risk of bias in individual studies when interpreting/ discussing the results of the review?”, “did the review authors report on the sources of funding for the studies included in the review?”, and “if meta-analysis was performed, did the review authors assess the potential impact of risk of bias in individual studies on the results of the meta-analysis or other evidence synthesis?” The interrater reliability of the methodological quality assessment was high (κ≥0.8).

### ROBIS Assessment

Multimedia Appendix 3 summarizes the risk of bias using ROBIS. 40% (5/12) of the studies had a low risk of bias, 20% (2/12) had an unclear risk of bias, and 40% (5/12) had a low risk of bias. The domains related to “data collection” and “synthesis and findings” had the highest risk of bias. On the other hand, the domain related to “eligibility criteria” had the lowest risk of bias. The interrater reliability of the risk of bias was high (k>0.8).

### PAGAC Grades of Evidence

The grades of evidence were classified as not assignable, limited, moderate, or strong according to the PAGAC. The level of evidence was limited to moderate among the studies. Most reviews evaluated a heterogeneous population of individuals, which limited the applicability and generalizability of the results. The domain “quantity and consistency” was affected because some studies had inconsistency in the direction of or the effect size itself (Multimedia Appendix 4).

### GROOVE Analysis

A total of 39 primary studies were identified across all systematic reviews and meta-analyses, 24 of which were distinct studies. The overall overlap in the matrix of evidence was moderate (CCA=32.5%) and it remained moderate (CCA=15.63%) after adjusting for the chronological structural missingness. Multimedia Appendix 5 shows a graphical representation of the GROOVE results.

### Telomere Length

**Figure 2.**
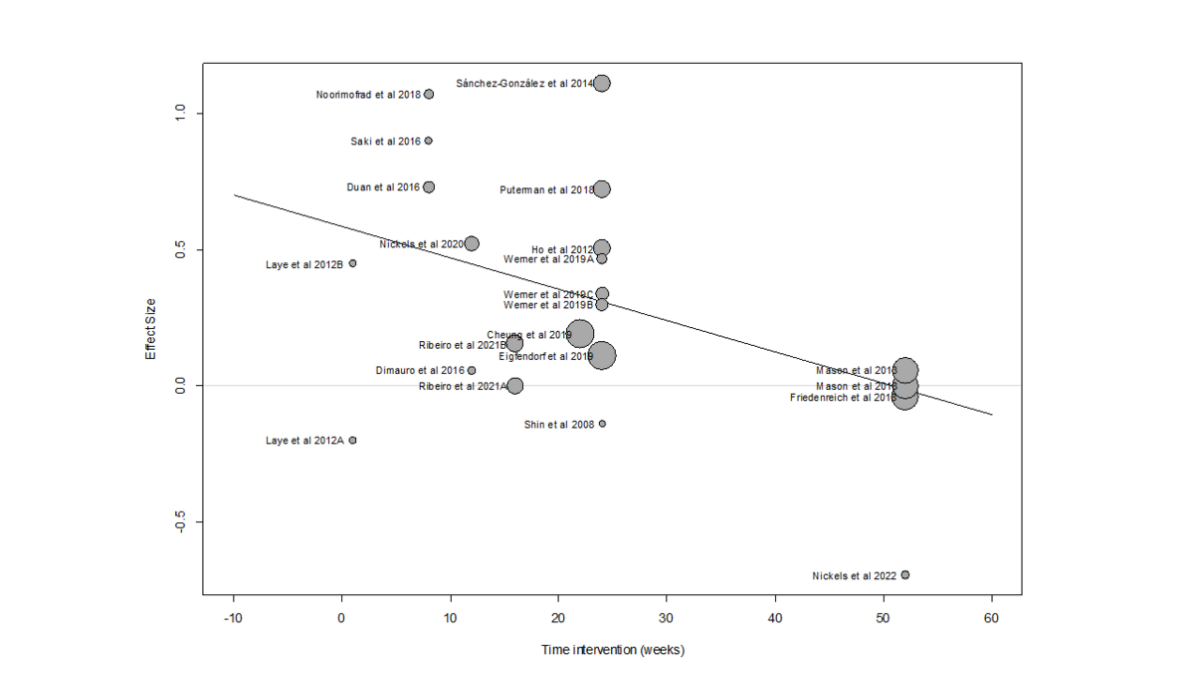
Scatterplot of primary studies including effect size and number of weeks of intervention.

The number of studies in the systemic reviews that were finally integrated in the quantitative (meta-analytic analysis) was 22. The estimated effect size was 0.28 (95% CI 0.118-0.439), with a heterogeneity test value (Q) of 43.08 (*P*=.003). The *I*^2^ coefficient was 51% and the prediction interval was −0.26 to 0.81. The number of weeks of intervention explained part of this heterogeneity (Q_B_=7.54; *P*=.006). This factor explained 20% of the overall heterogeneity. Figure 2 shows that studies where the number of weeks of intervention was greater than 30 had smaller effect sizes. In fact, if the intervention time was categorized into <30 and ≥30 weeks, Q_B_ was 11.64 (*P*<.001).

**Figure 3.**
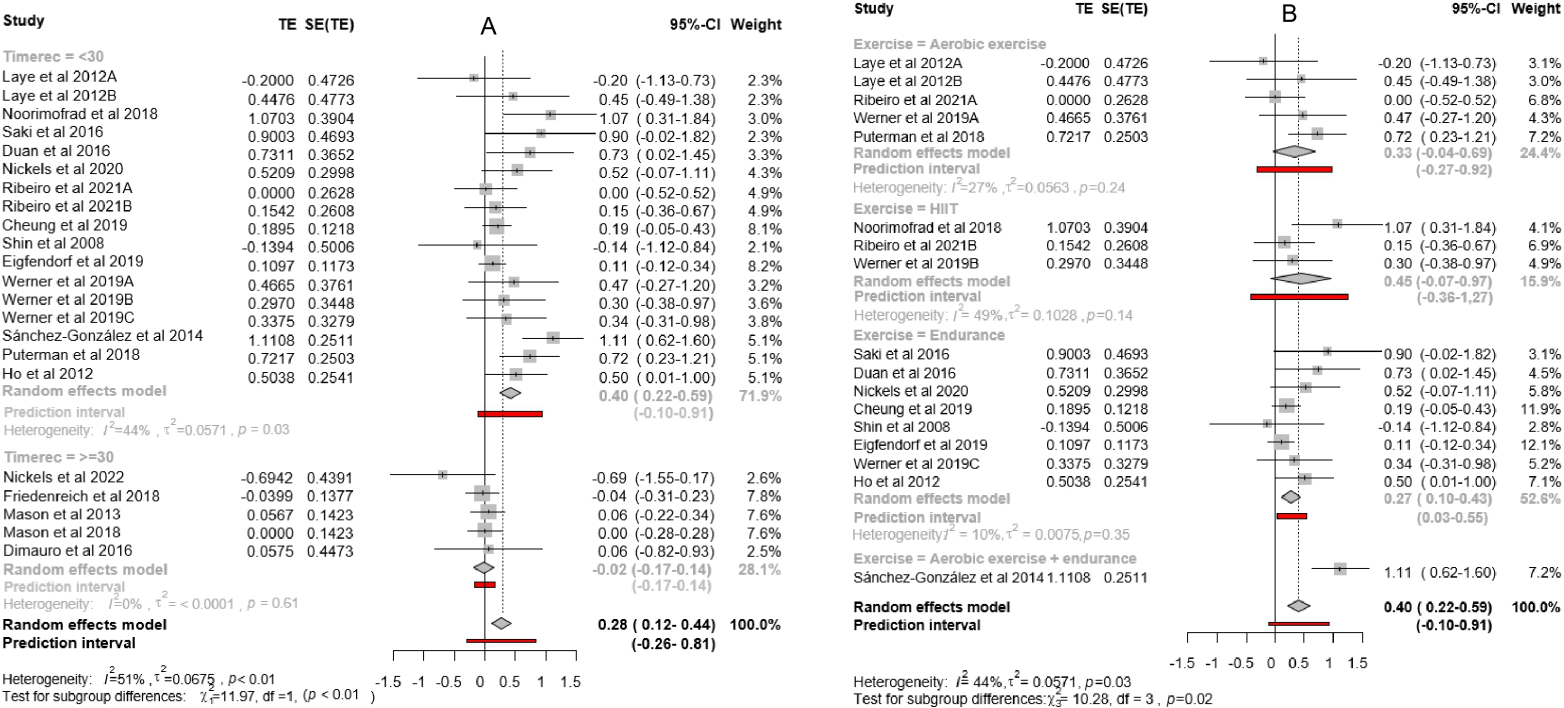
(A) Forest plot of subgroups of primary studies defined by length of intervention. (B) Forest plot of subgroups of primary studies defined by type of exercise. TE: treatment effect.

Figure 3A shows that the effect size was 0.39 for studies with an intervention length of less than 30 weeks, and −0.01 for those with interventions longer than 30 weeks. There was no significant difference in the average number of weekly sessions between these two groups (3.3 vs 3 sessions, respectively). Interestingly, the first group presented a statistically significant heterogeneity (*I*^2^=41%), whilst the second, despite having a large range of outcomes, did not. Since heterogeneity was high in the group of studies with shorter interventions (<30 wk), it was analyzed with respect to other variables. The factor that better explained this heterogeneity was the type of physical exercise defined by the 4 categories (subgroups) as shown in Figure 3 (Q_B_=11.15, *P*=.01). One subgroup was comprised of just 1 study that included a combination of aerobic exercise with endurance training and achieved a large effect size. The other 3 subgroups did not have a statistically significant heterogeneity between them, although the subgroup of studies defined by an intervention based on HIIT, which alternates short periods of intense anaerobic exercise with brief recovery periods, had a marked *I*^2^ value (49%). The HIIT subgroup only included 3 studies, one of which had a very high effect size compared to the other two. When we compared the effect sizes of the 3 types of noncombined physical exercises, the value was small for aerobic exercise and endurance training, and moderate for HIIT.

The contour-enhanced funnel plot (Multimedia Appendix 6) did not identify publication bias. Many of the effect sizes were not significant, and at the top of the funnel, there was some asymmetry, which could be due to the heterogeneity of results. The nonpresence of publication bias was confirmed with the Egger method (*P*=.10). The Doi plot and LFK index also found a weak asymmetry (Multimedia Appendix 7).

## Discussion

### Principal Findings

This umbrella review studied the effects of physical exercise on TL. The estimated overall effect size indicated a small-moderate positive impact of exercise on TL. However, we found some discrepancies in the results across studies that may be explained by several methodological and contextual reasons, including different methodologies for measuring TL, such as quantitative polymerase chain reaction and terminal restriction fragment assays, that can contribute to inconsistencies in TL calculations. In this regard, Smith et al [47] pointed out that intraindividual TL can vary significantly depending on the lab technique used to measure it. Differences in study populations could also be a factor responsible for disparities in results. Demographic factors, such as age, sex, ethnicity, and health status, can influence the relationship between exercise and TL. For instance, LaRocca et al [48] and Puterman et al [21] suggest that the benefits of exercise on TL may be more pronounced in older adults and in those with higher stress levels. In addition, the duration and intensity of the intervention could be another confounding factor. Our findings suggest that interventions longer than 30 weeks tend to show a lesser positive impact on TL. This could be due to a ceiling effect where additional benefits of exercise do not translate into further increase of TL. This is consistent with Werner et al [29], who observed that the beneficial effects of exercise on TL are more evident in the early phases of physical exercise programs.

The high heterogeneity in study designs, including interventions, participant adherence, and control measures, could have also contributed to different outcomes. In fact, Denham et al [23] and Puterman et al [21] have already highlighted the importance of these methodological issues when interpreting TL results. Studies such as Cherkas et al [20] had found that vigorous aerobic physical activity was associated with longer telomeres in adults, which is in line with our finding of a positive effect of aerobic exercise, especially when combined with endurance training, on TL. However, a systematic review by Du et al [49] did not identify a significant association between aerobic exercise and TL in younger populations, suggesting that age may be an important moderating factor. Denham et al [23] found mixed results related to endurance physical training. Our review also found significant variation in the effects of noncombined endurance training, which suggests that factors such as the intensity and duration of this type of physical exercise may influence the magnitude of the effect on TL. Finally, HIIT had previously shown a significant positive effect on TL [29]. Our review results are consistent with this; nonetheless, the low number of HIIT studies and variety of designs limit generalizability.

### Biological Understanding of the Effect of Exercise on TL

#### Reduction of Oxidative Stress and Inflammation

Oxidative stress and chronic inflammation are 2 major factors contributing to telomere erosion [50,51]. Studies have shown that regular exercise can mitigate these factors [52-54]. Acute and chronic physical activity have different effects on oxidative stress. While acute exercise triggers the production of reactive oxygen and nitrogen species, leading to oxidative stress, regular exercise training enhances the body’s endogenous antioxidant system, offering protection against the harmful effects of oxidative damage [55]. This antioxidant effect protects cells from oxidative damage, which in turn preserves the integrity of telomeres. In addition, exercise reduces the levels of inflammatory markers such as C-reactive protein and tumor necrosis factor-alpha, which can protect telomeres from inflammation-induced damage [56].

#### Increase in Telomerase Activity

Telomerase is a crucial enzyme for maintaining TL, as it adds repetitive sequences to the end of chromosomes. Research suggests that exercise can increase telomerase activity. Ornish et al [57] demonstrated that an intensive lifestyle change program, which included regular exercise, significantly increased telomerase activity in men with prostate cancer. This finding suggests that exercise can directly influence telomere biology by activating telomerase, which helps maintain TL and protects cells from premature aging. In addition, a systematic review with meta-analysis published by Denham et al [23] concludes that regular exercise may attenuate telomere attrition through a telomerase-dependent mechanism and ultimately prolong lifespan and longevity. Furthermore, Ludlow et al [28] found that moderate physical activity levels are associated with increased telomerase activity in leukocytes, indicating that regular exercise not only improves overall health, but also acts at a molecular level to protect cells from premature aging. These studies highlight the importance of exercise continuity and intensity in maximizing the benefits related to telomerase activity and cellular longevity.

#### Improvement of Cardiovascular Capacity and Metabolic Health

Improved cardiovascular capacity and metabolic health are also associated with longer telomeres. Puterman et al [21] found that better aerobic capacity and higher insulin sensitivity are associated with longer telomeres. These cardiovascular and metabolic benefits reduce oxidative stress and inflammation, creating a healthier cellular environment that protects TL. Regular exercise enhances cardiovascular system efficiency, increases blood circulation, and facilitates the delivery of oxygen and nutrients to cells, which can contribute to the preservation of telomeres.

### Strengths and Limitations

We present a comprehensive analysis of systematic reviews and meta-analyses of the effect of physical exercise on TL, providing a broad and robust synthesis of the available evidence. The methodology used to assess the quality of studies and the application of standardized tools for evaluating the risk of bias and heterogeneity strengthens the validity of our results. However, our work has some limitations. The methodological quality of the reviews included in this analysis varies, with many of them classified as critically low. Also, the heterogeneity in study designs, samples, and interventions makes difficult to generalize the results, even after the use of a random effects model to integrate outcomes. Furthermore, some meta-analyses reported results only using postintervention measures, whilst others used postintervention differences adjusted for preintervention measures; this may lead to variations in effect sizes.

### Clinical Implications

The implementation of exercise programs into clinical practice could be an effective strategy to limit telomere shortening, promoting healthy aging and reducing the incidence of age-related diseases. HIIT and the combination of aerobic exercise with endurance training may be promising interventions in this regard but still require to be further tested in high-quality studies. In addition, physical exercise should be tailored to each individual’s physical capability and health condition to maximize cellular health benefits. Implementing physical exercise programs will also require ongoing health education and monitoring to ensure adherence to and adjustment of interventions based on observed outcomes. Future studies should investigate sex and ethnic TL differences in response to various types of physical exercise. This could help personalize recommendations and optimize benefits on cellular health and aging.

### Conclusions

Our umbrella review and meta-analysis identified a small-moderate positive effect of physical exercise on TL, which seems to be influenced by the duration and type of physical exercise. In health care systems, the implementation of evidence-based physical exercise, training programs, and/or recommendations tailored to each individual might be a valuable preventive strategy for a healthy aging. More studies, with larger sample sizes, testing the impact of standardized, evidence-based physical exercise interventions on TL are warranted.

## Supplementary material

10.2196/64539 Multimedia Appendix 1 Database formulas during literature search.

10.2196/64539 Multimedia Appendix 2 Risk of bias.

10.2196/64539 Multimedia Appendix 3 Graphical representation of the overlap of primary studies between reviews.

10.2196/64539 Multimedia Appendix 4 Contour-enhanced funnel plot of the primary studies included in the meta-analysis.

10.2196/64539 Multimedia Appendix 5 Doi plot and LFK index of the primary studies included in the meta-analysis.

10.2196/64539 Multimedia Appendix 6 Quality assessment scores (A Measurement Tool to Assess Systematic Reviews [AMSTAR-2]).

10.2196/64539 Multimedia Appendix 7 Summary of findings and quality of evidence (Physical Activity Guidelines Advisory Committee Grading Criteria [PAGAC]).

10.2196/64539 Checklist 1 PRISMA (Preferred Reporting Items for Systematic Reviews and Meta-Analyses) checklist.

## Abbreviations

AMSTAR-2: A Measurement Tool to Assess Systematic Reviews
CCA: corrected covered area
GROOVE: Graphical Representation of Overlap for OVErviews
HIIT: high-intensity interval training
PAGAC: Physical Activity Guidelines Advisory Committee Grading Criteria
PICOS: Population, Intervention, Control, Outcomes, and Study design
PRISMA: Preferred Reporting Items for Systematic Reviews and Meta-Analyses
ROBIS: Risk Of Bias in Systematic reviews
TL: telomere length
