# Supplementary material for: Effect of Physical Exercise on Telomere Length: Umbrella Review and Meta-Analysis
Source: JMIR Aging. 2025 Jan 10;8:e64539. doi: 10.2196/64539 (PMC11755188; doi:10.2196/64539)
Supplement: Multimedia Appendix 1 [file aging-v8-e64539-s001.docx]

| **Appendix 1:** Database formulas during literature search (19/01/2024) |
| --- |
| **PubMed Search Formula: 521**  ("telomere" OR "telomere length") AND ("physical activity" OR "exercise" OR "aerobic exercise" OR "acute exercise" OR "sports" OR "physical fitness" OR "exercise therapy" OR "resistance training" OR "muscle training" OR "exercise training" OR "physical exercise") |
| **Cochrane Library Search Formula: 128**  ("telomere" OR "telomere length") in Title Abstract Keyword AND ("physical activity" OR "exercise" OR "aerobic exercise" OR "acute exercise" OR "sports" OR "physical fitness" OR "exercise therapy" OR "resistance training" OR "muscle training" OR "exercise training" OR "physical exercise") in Title Abstract Keyword |
| **SCOPUS Search Formula: 884**  TITLE-ABS-KEY ("telomere" OR "telomere length") AND ("physical activity" OR "exercise" OR "aerobic exercise" OR "acute exercise" OR "sports" OR "physical fitness" OR "exercise therapy" OR "resistance training" OR "muscle training" OR "exercise training" OR "physical exercise") |
| **WOS Search Formula: 889** |

("telomere" OR "telomere length") AND ("physical activity" OR "exercise" OR "aerobic exercise" OR "acute exercise" OR "sports" OR "physical fitness" OR "exercise therapy" OR "resistance training" OR "muscle training" OR "exercise training" OR "physical exercise")

**Embase Search Formula: 1206**

("telomere" OR "telomere length") AND ("physical activity" OR "exercise" OR "aerobic exercise" OR "acute exercise" OR "sports" OR "physical fitness" OR "exercise therapy" OR "resistance training" OR "muscle training" OR "exercise training" OR "physical exercise")
