# Supplementary figures and images for: Effect of Physical Exercise on Telomere Length: Umbrella Review and Meta-Analysis
Source: JMIR Aging. 2025 Jan 10;8:e64539. doi: 10.2196/64539 (PMC11755188; doi:10.2196/64539)

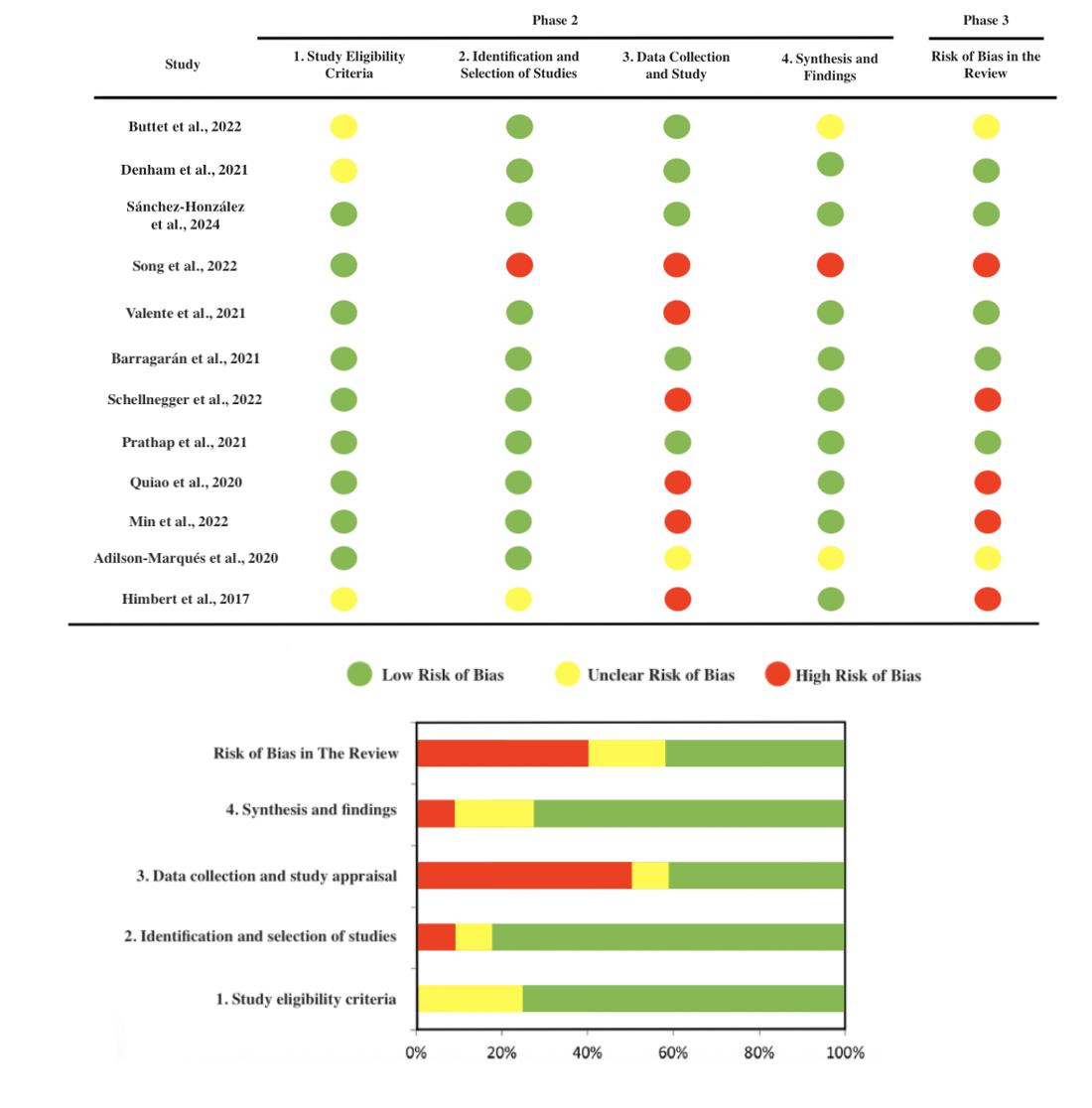

Supplement: Multimedia Appendix 2 [file aging-v8-e64539-s002.png]

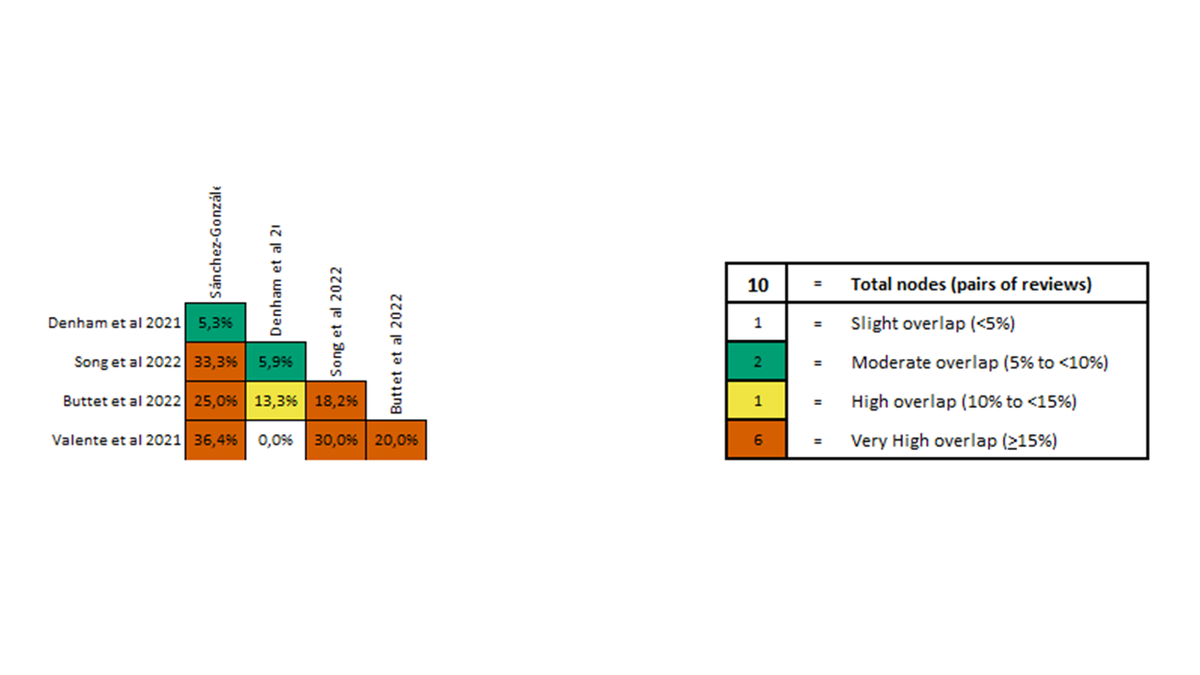

Supplement: Multimedia Appendix 3 [file aging-v8-e64539-s003.png]

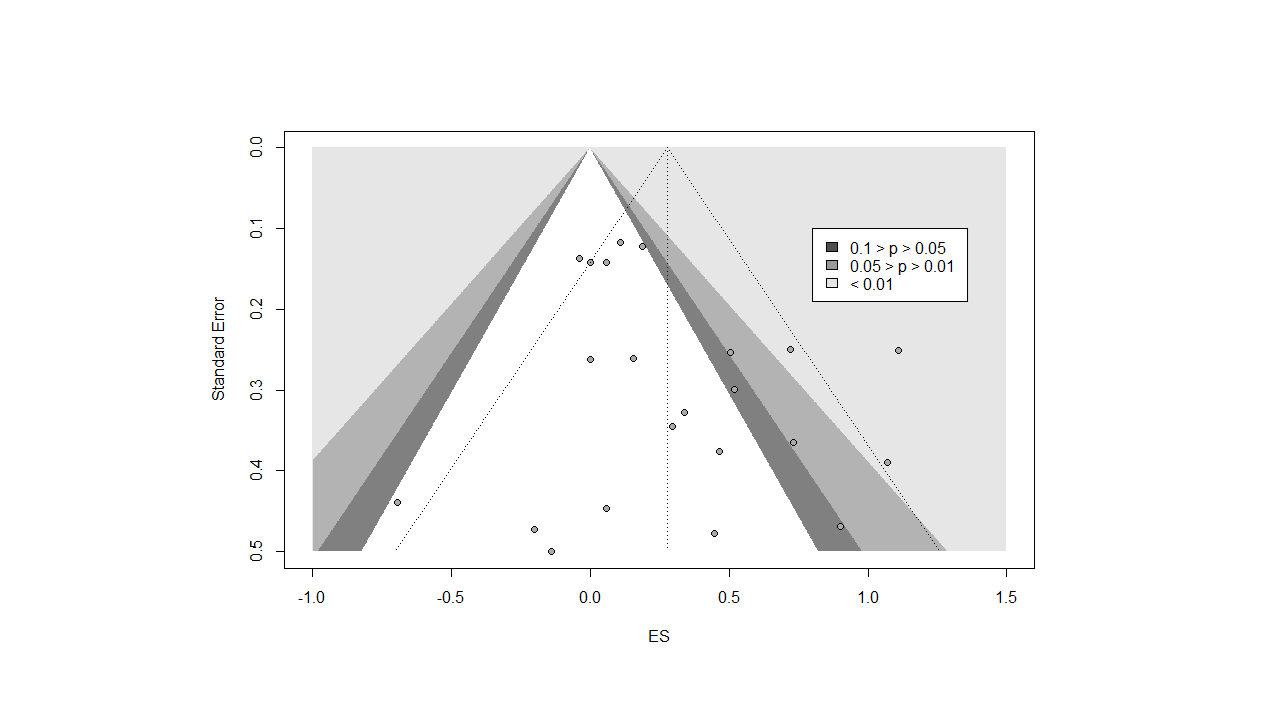

Supplement: Multimedia Appendix 4 [file aging-v8-e64539-s004.png]

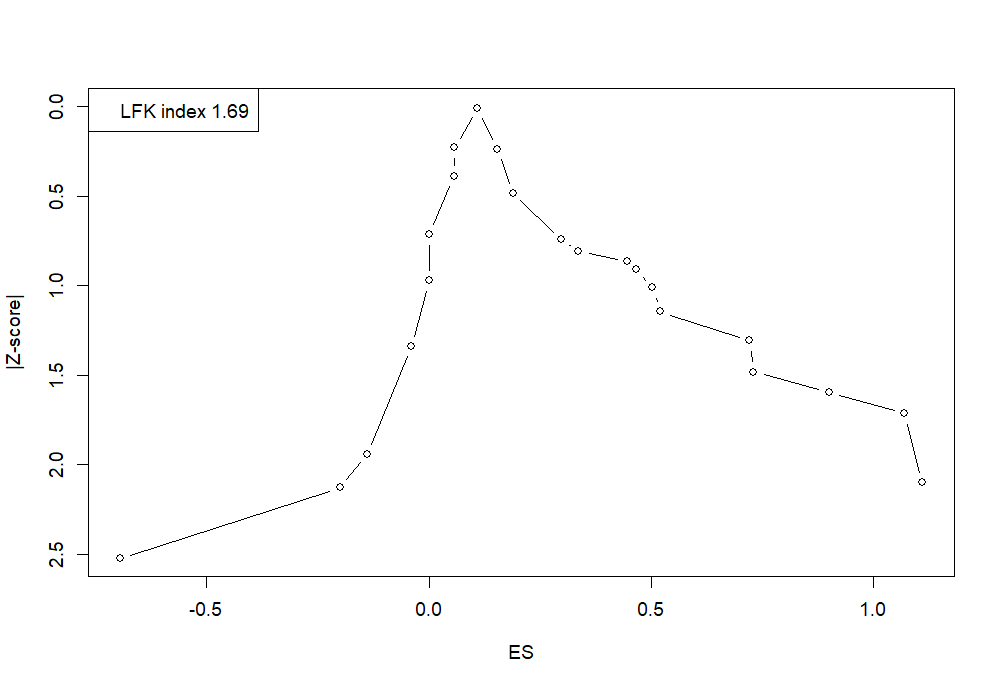

Supplement: Multimedia Appendix 5 [file aging-v8-e64539-s005.png]
