# Supplementary material for: Effect of Physical Exercise on Telomere Length: Umbrella Review and Meta-Analysis
Source: JMIR Aging. 2025 Jan 10;8:e64539. doi: 10.2196/64539 (PMC11755188; doi:10.2196/64539)
Supplement: Multimedia Appendix 7 [file aging-v8-e64539-s007.docx]

**Supplementary Material Table 2. Summary of findings and quality of evidence (PAGAC).**

| Study | Applicability | Generalizability | RoB or study limitations | Quantity and consistency | Magnitude and precision of effect | Grade of evidence |
| --- | --- | --- | --- | --- | --- | --- |
| Song et al 2022 | Moderate | Limited | Moderate | Limited | Limited | Limited |
| Buttet el al 2022 | Strong | Strong | Moderate | Moderate | Moderate | Moderate |
| Sánchez-González et al 2024 | Strong | Strong | Strong | Moderate | Moderate | Moderate |
| Valente et al 2021 | Moderate | Moderate | Moderate | Limited | Moderate | Moderate |
| Denham et al 2021 | Limited | Limited | Moderate | Moderate | Moderate | Limited |
| Barragán et al 2021 | Limited | Moderate | Strong | Moderate | NA | Moderate |
| Schellnegger et al 2022 | Moderate | Moderate | Limited | Moderate | NA | Moderate |
| Prathap et al 2021 | Moderate | Moderate | Strong | Limited | NA | Limited |
| Quiao et al 2020 | Limited | Limited | Limited | Limited | NA | Limited |
| Min et al 2022 | Strong | Strong | Limited | Limited | NA | Limited |
| Adilson-Marques et al 2020 | Limited | Limited | Moderate | Limited | NA | Limited |
| Himbert et al 2017 | Limited | Limited | limited | Limited | NA | Limited |
| Criteria: Strong; Moderate; Limited; Not Assignable. | | | | | | |
